# The dcGO Domain-Centric Ontology Database in 2023: New Website and Extended Annotations for Protein Structural Domains

**DOI:** 10.1016/j.jmb.2023.168093

**Published:** 2023-04-13

**Authors:** Chaohui Bao, Chang Lu, James Lin, Julian Gough, Hai Fang

**Affiliations:** 1 **Shanghai Institute of Hematology,** State Key Laboratory of Medical Genomics, National Research Center for Translational Medicine at Shanghai, Ruijin Hospital, Shanghai Jiao Tong University School of Medicine, Shanghai 200025, China; 2 **MRC Laboratory of Molecular Biology,** Francis Crick Avenue, Cambridge Biomedical Campus, Cambridge CB2 0QH, UK; 3 **MRC London Institute of Medical Sciences,** Imperial College London, London W12 0HS, UK; 4 **High Performance Computing Center,** Shanghai Jiao Tong University, Shanghai 200240, China

**Keywords:** protein structural domains, ontologies, annotations, enrichment analysis, computational resources

## Abstract

Protein structural domains have been less studied than full-length proteins in terms of ontology annotations. The dcGO database has filled this gap by providing mappings from protein domains to ontologies. The dcGO update in 2023 extends annotations for protein domains of multiple definitions (SCOP, Pfam, and InterPro) with commonly used ontologies that are categorised into functions, phenotypes, diseases, drugs, pathways, regulators, and hallmarks. This update adds new dimensions to the utility of both ontology and protein domain resources. A newly designed website at http://www.protdomainonto.pro/dcGO offers a more centralised and user-friendly way to access the dcGO database, with enhanced faceted search returning term- and domain-specific information pages. Users can navigate both ontology terms and annotated domains through improved ontology hierarchy browsing. A newly added facility enables domain-based ontology enrichment analysis.

## Graphical abstract

**Graphic abstract:**
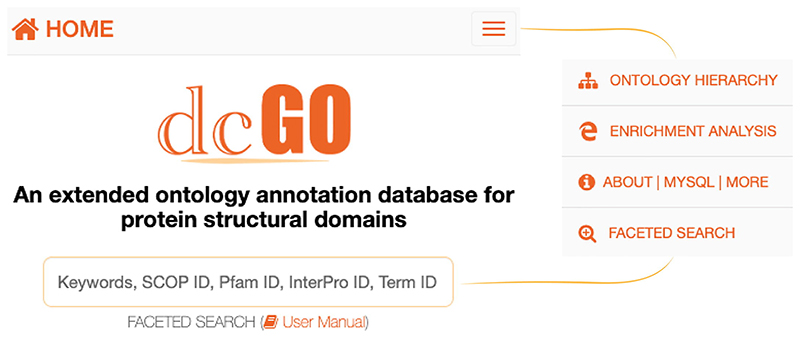

## Introduction

Computational prediction of protein structures has become feasible,^1^ but most available protein sequences lack biological annotations.^2^ Protein structural domains have received less attention than full-length proteins in terms of ontology annotations, such as annotations using Gene Ontology (GO).^3^ To resolve this gap, about ten years ago we developed a domain-centric method^4^ to create the dcGO database,^5^ an ontology resource that provides annotations for protein structural domains. A growing number of ontologies have been created to annotate full-length proteins; however, there is a significant need for using ontologies to annotate protein domains. Domain-centric ontology annotation resources are essential since protein domains often act as the functional units of proteins and haven been shown to be useful in protein function prediction^6,7^ and more recently in hypothesis-free phenotype prediction.^8^

Over time, dcGO has evolved to support domain-centric annotations not only for protein domains taken from the structural classification of protein (SCOP) at both the superfamily and family levels,^9^ but also for domains from Pfam^10^ and InterPro.^11^ In parallel with the growth in ontology knowledge-bases, these domain-centric annotations are available across various knowledge contexts, ranging from functions and pathways to phenotypes and diseases, and even drugs. Systematic mappings from protein domains to ontology terms, via dcGO, maximise the utility of both ontology and domain resources.

**Figure 1:**
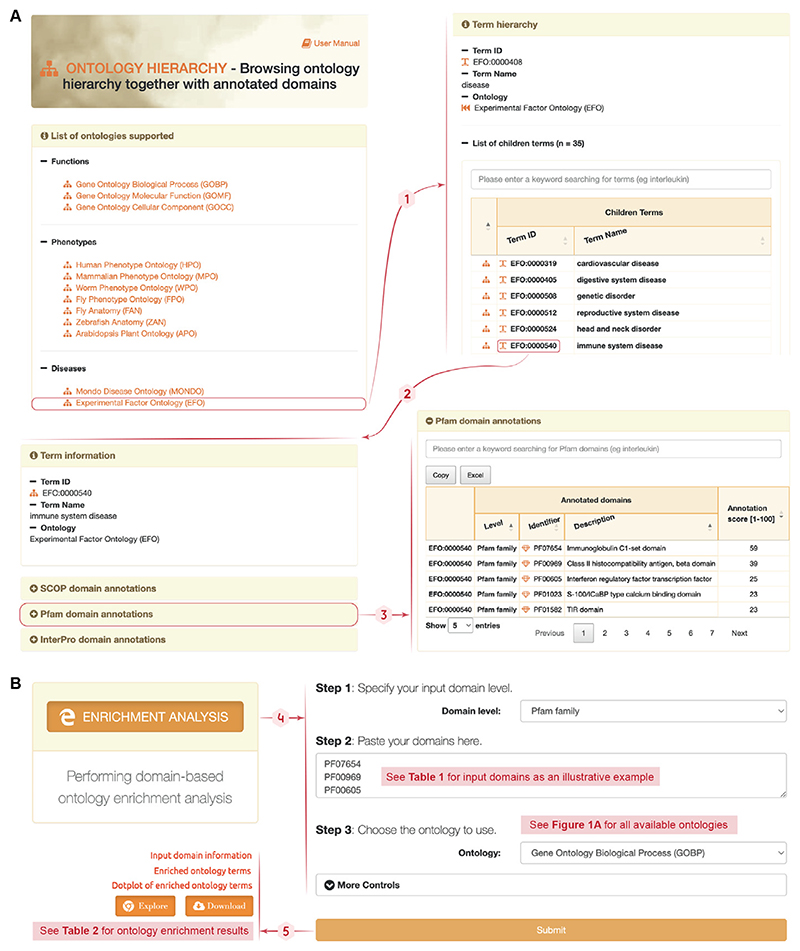
Content and website of the dcGO database in 2023. **(A)** The content. *Top*: ontologies are categorised into functions, pathways, phenotypes, diseases, drugs, regulators, and hallmarks. *Bottom*: a treemap summarises the database content. Each box represents an ontology and is color-coded by the total number of annotations per ontology. The treemap describes numbers on annotations, ontology terms, and protein domains of different definitions (i.e., SCOP, Pfam, and InterPro). SF, SCOP superfamilies; FA, SCOP families. **(B)** The website. It includes interfaces for browsing the ontology hierarchy and annotated domains, performing domain-based ontology enrichment analysis, providing the help on database access, and using the faceted search to explore the dcGO resource. Notably, the faceted search enables simultaneous search for protein domains (of different definitions) and ontology terms (of various categories).

Since our previous publications closely related to dcGO,^4,5,12,13^ we have continued to expand ontologies and domains, and considerably, we have redesigned a new website (Figure 1). The website includes a booklet-style user manual and features enhanced faceted search (augmenting search results with a faceted navigation system,^14^ improved ontology hierarchy browsing, and domain-based ontology enrichment analysis. All these improvements represent the dcGO database update in 2023, which we will describe in detail in the following sections.

## Materials and methods

### The dcGO building method

The building method has evolved over time and can be simplified into the following steps:

(i) Prepare a correspondence matrix^5,6^ that records the observed number of proteins (i.e. matrix entries) with structural domains (in columns) and ontology terms (in rows).
(ii) Deduce associations/annotations between domains and terms from the corresponding matrix using Fisher’s exact test. The annotation significance is measured by false discovery rate (FDR) with Benjamini-Hochberg corrections for multiple hypothesis testing,^15^ and the annotation strength is quantified by a hypergeometric distribution-based score (or ‘annotation score’) rescaled into the 1–100 range.
(iii) Propagate domain-centric annotations to all ancestor terms (along with annotation scores) according to the ‘True Path Rule’, which respects the directed acyclic graph of an ontology (e.g. GO).^16^ This rule ensures that a protein domain annotated to a term must also be annotated by its top-level parent terms in paths towards the ontology root.^5^

In summary, the dcGO building method takes as inputs ontology terms attached to proteins and the domain composition of proteins, and then statistically infers mappings from protein domains to ontology terms within a probabilistic framework. For further details, users are referred to our previous publications on the method.^4,5^ In this 2023 update, the method has been applied to almost all commonly used ontologies for protein domains of different definitions, which are described in greater detail below.

### Protein domains of different definitions

Presently, the dcGO database provides ontology annotations for protein domains taken from SCOP,^9^ Pfam,^10^ and InterPro^11^ (Figure 1(A)). Annotations are supported for SCOP at both the superfamily and family levels. SCOP domains are classified into a superfamily if there exists structure, sequence, and function evidence for a common evolutionary ancestor. Superfamilies can be further divided into families based on high sequence similarity or related function. In addition to SCOP, ontology annotations have also been extended to approximately 1,000 Pfam domains and around 800 Inter-Pro domains, two popular protein family resources.

### Commonly used ontologies

The dcGO update in 2023 now conveniently organises ontologies into seven broad categories (Figure 1(A)):

(i) Functions: GO^17^ (accessed in October 2022), which includes GO Biological Process (GOBP), GO Molecular Function (GOMF), and GO Cellular Component (GOCC).
(ii) Phenotypes: This category includes Human Phenotype Ontology (HPO)^18^ (June 2022 release), Mammalian Phenotype Ontology (MPO)^19^ (accessed in July 2022), and other phenotype and anatomy ontologies for model organisms such as WormBase^20^ (WS284 release), FlyBase^21^ (6.48 release), ZFIN^22^ (accessed in July 2022), and TAIR^23^ (accessed in July 2022).
(iii) Diseases: This category includes Mondo Disease Ontology (MONDO) that harmonises disease definitions across the world^24^ (v2023-01-04 release), and Experimental Factor Ontology (EFO) used to annotate genome-wide association study (GWAS) disease traits^25^ (3.44.0 release).
(iv) Drugs: That is, druggable categories from DGIdb^26^ (2022-Feb release) and target tractability buckets (Bucket) from Open Targets^27^ (22.06 release).
(v) Pathways: This category primarily includes sources from KEGG^28^ (103.0 release), REACTOME^29^ (version 81 release), PANTHER^30^ (17.0 release), WikiPathways^31^ (July 2022 release), and MitoPathways from MitoCarta^32^ (MitoCarta3.0 version).
(vi) Regulators: That is, ENRICHR Consensus TFs^33^ (accessed in July 2022) and TRRUST^34^ (2018.04.16 release).
(vii) Hallmarks: Molecular signature hallmarks from MSigDB^35^ (v7.5.1 release).

### The dcGO website

The website has been revamped using the Mojolicious Perl real-time web framework (https://mojolicious.org) and Bootstrap (https://getbootstrap.com) to support a mobile-first and responsive web experience for all major browsers and devices. To enable faceted search, the website uses the typeahead JavaScript library (https://twitter.github.io/typeahead.js), which includes a suggestion engine for queries (ontology terms or protein domains) and a user interface view for rendering suggestions and handling hyperlinks from search results. Enrichment results from domain-based enrichment analysis are rendered using the bookdown R package (https://bookdown.org), which generates self-contained dynamic HTML files in the enrichment results page. The source code for the dcGO website is made available at GitHub (https://github.com/hfang-bristol/dcGO).

## Results and discussion

### Faceted search as a hub to explore the dcGO resource

The dcGO website offers a powerful faceted search (Figure 1(B)) that allows users to perform multiple tasks with hyperlinks from the search 3 results. This is enabled using a flexible JavaScript library to create robust typeaheads (see Materials and Methods). The search engine supports fulltext queries for protein domains and ontology terms. When users search for an ontology term, the results are hyperlinked to a term-specific page, which displays a table of annotated domains. Similarly, when searching for a particular protein domain, the results are hyperlinked to a domain-specific page, which displays a table of ontology terms used to annotate that protein domain. These tabular displays include annotation scores that quantify the support for annotations between domains and terms. By clicking on the hyperlinks provided, users can easily switch between domain-specific and term-specific pages. In conclusion, the faceted search not just provides search results but also interconnects all database contents, enabling users to perform integrated mining of the dcGO resource.

### Browsing ontology hierarchy and annotated domains

The dcGO website features the ‘Ontology Hierarchy’ navigation that allows users to browse ontology hierarchies.Figure 1(A) summarises the ontologies currently supported in the database. As before, the most abundant annotations are seen for ontologies related to functions and phenotypes. The least abundant domain-centric annotations are seen for mitochondrial pathways, which have recently been added to the dcGO database. The ontology hierarchy has a node for each term and directed edges linking it to its children nodes. All direct children of the current node are listed underneath, allowing users to browse the hierarchy in a downward direction. In addition to the hierarchy itself, the toggle panels for domain-centric annotations are also displayed separately for SCOP, Pfam, and InterPro.

**Figure 2:**
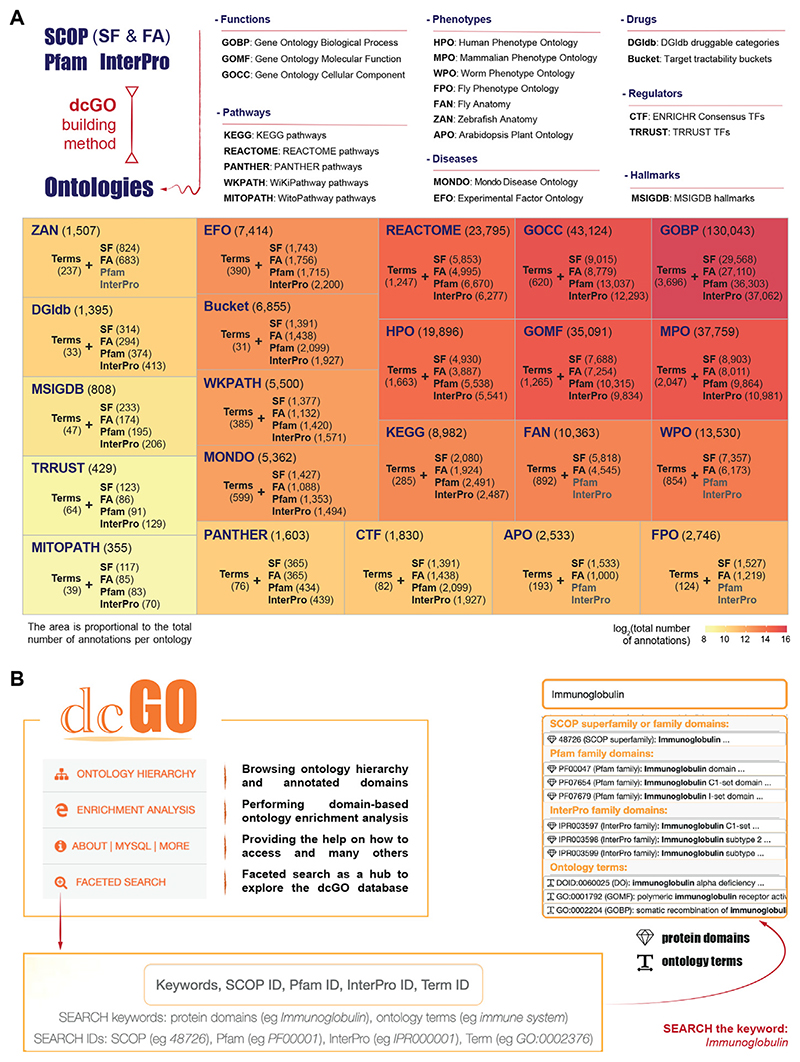
Illustrating how to use the resource via ontology hierarchy browsing and domain-based ontology enrichment analysis. The integers in the hexagons denote sequential clicks. **(A)** Interfaces for exploring the ontology hierarchy and annotated domains. *Top-left*, the hierarchy page lists all supported ontologies, including Experimental Factor Ontology (EFO). *Top-right*: the EFO term ‘disease’ (EF0.0000408) and its child terms. Each child term provides a hyperlink to the hierarchy page and a hyperlink to the term-specific page. *Bottom*: the term-specific page for the child term ‘immune system disease’ (EF0.0000540), which lists the annotated domains separately for SCOP, Pfam and InterPro; for example, Pfam domain annotations (also listed in Table 1). **(B)** Domain-based ontology enrichment analysis for identifying enriched ontology terms from user-input protein domains. *Left*, the user-request interface, which takes a list of user-input protein domains and their matched domain type, available ontologies, and additional parameters for more control over the enrichment analysis and results. Enrichment results include a table (see Table 2) and a dot plot, all embedded into a self-contained dynamic HTML file available for exploration and download.

To illustrate how users can access ontologies and annotated domains, we take as an exemplar the EFO,36 a newly added ontology in the dcGO database that enables domain-centric annotations with GWAS disease traits (Figure 2(A)). The hierarchy roots of all supported ontologies in dcGO can be found on the landing page, including the EFO root term ‘disease’ (EFO:0000408). This root term is hyperlinked to its detailed hierarchy page (Click 1 of Figure 2(A)), displaying its 35 child terms in a table. In this table, each child term [such as ‘immune system disease’ (EFO:0000540)] provides a hyperlink to both the hierarchy page and the term-specific page (Click 2 of Figure 2(A)). The term-specific page displays a table of annotated domains, grouped separately by SCOP, Pfam, and InterPro. For example, a total of 33 Pfam domains are annotated to the ‘immune system disease’ term, and these annotations are sorted by their annotation scores (Click 3 of Figure 2(A); also see Table 1). Users can explore these annotations using hyperlinks that lead to the domain-centric pages. In summary, the ontology hierarchy interfaces offer a more integrated and cohesive way to navigate ontology terms and annotated domains.

### A new facility supporting domain-based ontology enrichment analysis

The dcGO resource provides a unique reference knowledgebase for domain-centric ontology annotations, and a new facility has been developed to perform enrichment analysis for user-input protein domains. This facility enables the identification of enriched ontology terms, a feature not available in other web-based enrichment analysis tools (for example, DAVID web server for enrichment analysis focusing on genes/proteins^37^). The user-request interface (Click 4 of Figure 2(B) allows users to input a list of protein domains and their matched domain type, as well as select available ontologies (organised by category; see Figure 1 (A)). Additional parameters can be specified to control the analysis and results. The interface provides an example showcase (that is, 33 Pfam domains described above in Figure 2(A)). In the enrichment results page, the enriched ontology terms are presented in an interactive table, along with the significant information such as Z-scores and FDR, and member domains that overlap with the input domains (Click 5 of Figure 2(B); Table 2). The results are also illustrated in the *‘Dotplot of enriched ontology terms’* tab, which shows the top five terms with their respective Z-scores and FDR. All enrichment results are embedded into a self-contained dynamic HTML file, which can be downloaded and explored interactively in a new browser window, making it easy for users to explore the results further.

## Conclusion

In this updated version of the dcGO resource, our continued focus is on providing systematic mappings from protein domains to ontologies. We are excited to introduce a new website with enhanced data analyses and a unique facility for identifying ontology knowledge enrichments from the perspective of domain-centric annotations. Our commitment to updating the resource twice a year includes integrating information from our previously established resources such as XGR,^38^ SUPERFAMILY,^39^ and Priority index.^40–42^ Looking to the future, we are also excited to explore the potential of large language models^43^ in generating domain-centric ontologies, following their success in generating functional protein sequences.^44^

## Figures and Tables

**Table 1:** List of Pfam domains annotated to the EFO term ‘immune system disease’.

Identifier	Description	Annotation score [1–100]
PF07654	Immunoglobulin C1-set domain	59
PF00969	Class II histocompatibility antigen, beta domain	39
PF00605	Interferon regulatory factor transcription factor	25
PF01023	S-100/ICaBP type calcium binding domain	23
PF01582	TIR domain	23
PF00017	SH2 domain	22
PF00229	TNF (Tumour Necrosis Factor) family	22
PF00020	TNFR/NGFR cysteine-rich region	22
PF00048	Small cytokines (intecrine/chemokine), interleukin-8 like	19
PF01108	Tissue factor	18
PF00619	Caspase recruitment domain	17
PF00008	EGF-like domain	16
PF03770	Inositol polyphosphate kinase	16
PF01017	STAT protein, all-alpha domain	16
PF02864	STAT protein, DNA binding domain	16
PF02865	STAT protein, protein interaction domain	16
PF09294	Interferon-alpha/beta receptor, fibronectin type III	15
PF10401	Interferon-regulatory factor 3	15
PF00129	Class I Histocompatibility antigen, domains alpha 1 and 2	14
PF00178	Ets-domain	14
PF00993	Class II histocompatibility antigen, alpha domain	13
PF00001	7 transmembrane receptor (rhodopsin family)	12
PF00023	Ankyrin repeat	12
PF00656	Caspase domain	11
PF07686	Immunoglobulin V-set domain	11
PF02198	Sterile alpha motif (SAM)/Pointed domain	11
PF07714	Protein tyrosine and serine/threonine kinase	10
PF00018	SH3 domain	10
PF07716	Basic region leucine zipper	8
PF00170	bZIP transcription factor	8
PF00173	Cytochrome b5-like Heme/Steroid binding domain	8
PF00130	Phorbol esters/diacylglycerol binding domain (C1 domain)	5
PF00169	PH domain	2

**Table 2:** List of top 5 enriched GOBP terms.

Term ID	Term Name	Z-score	FDR	Num	Member domains
GO:0002376	immune system process	13.2	6.90E-17	22	PF00001, PF00008, PF00017, PF00018, PF00020, PF00048, PF00129, PF00130, PF00169, PF00229, PF00605, PF00619, PF00656, PF00969, PF00993, PF01108, PF01582, PF07654, PF07686, PF07714, PF07716, PF10401
GO:0048522	positive regulation of cellular process	9.19	3.30E-14	29	PF00001, PF00008, PF00017, PF00018, PF00020, PF00023, PF00048, PF00129, PF00130, PF00169, PF00170, PF00178, PF00229, PF00605, PF00619, PF00656, PF00969, PF00993, PF01017, PF01023, PF01582, PF02198, PF02864, PF02865, PF07654, PF07686, PF07714, PF07716, PF10401
GO:0002684	positive regulation of immune system process	12.3	7.70E-13	16	PF00001, PF00017, PF00018, PF00020, PF00048, PF00129, PF00130, PF00169, PF00229, PF00619, PF00969, PF00993, PF01582, PF07654, PF07686, PF07714
GO:0006952	defense response	11.4	9.60E-13	18	PF00001, PF00017, PF00018, PF00020, PF00048, PF00129, PF00605, PF00619, PF01017, PF01023, PF01108, PF01582, PF02864, PF02865, PF07654, PF07714, PF09294, PF10401
GO:0006950	response to stress	9.55	9.60E-13	24	PF00001, PF00008, PF00017, PF00018, PF00020, PF00023, PF00048, PF00129, PF00130, PF00169, PF00170, PF00605, PF00619, PF01017, PF01023, PF01108, PF01582, PF02864, PF02865, PF07654, PF07714, PF07716, PF09294, PF10401

## Data Availability

All dcGO data and online tools are provided to the public free of charge.
